# Basal Keratinocytes Contribute to All Strata of the Adult Zebrafish Epidermis

**DOI:** 10.1371/journal.pone.0084858

**Published:** 2014-01-06

**Authors:** Raymond T. H. Lee, P. V. Asharani, Thomas J. Carney

**Affiliations:** Discovery Research Division, Institute of Molecular and Cell Biology (IMCB), A*STAR (Agency for Science, Technology and Research), Singapore, Republic of Singapore; University of Queensland, Australia

## Abstract

The epidermis of terrestrial vertebrates is a stratified epithelium and forms an essential protective barrier. It is continually renewed, with dead corneocytes shed from the surface and replaced from a basal keratinocyte stem cell population. Whilst mouse is the prime model system used for epidermal studies, there is increasing employment of the zebrafish to analyse epidermis development and homeostasis, however the architecture and ontogeny of the epidermis in this system are incompletely described. In particular, it is unclear if adult zebrafish epidermis is derived entirely from the basal epidermal stem cell layer, as in the mouse, or if the most superficial keratinocyte layer is a remnant of the embryonic periderm. Furthermore, a relative paucity of cellular markers and genetic reagents to label and manipulate the basal epidermal stem cell compartment has hampered research. Here we show that the type I keratin, *krtt1c19e*, is a suitable marker of the basal epidermal layer and identify a *krtt1c19e* promoter fragment able to drive strong and specific expression in this cell type. Use of this promoter to express an inducible Cre recombinase allowed permanent labelling of basal cells during embryogenesis, and demonstrated that these cells do indeed generate keratinocytes of all strata in the adult epidermis. Further deployment of the Cre-Lox system highlighted the transient nature of the embryonic periderm. We thus show that the epidermis of adult zebrafish, as in the mouse, derives from basal stem cells, further expanding the similarities of epidermal ontogeny across vertebrates. Future use of this promoter will assist genetic analysis of basal keratinocyte biology in zebrafish.

## Introduction

All organisms exist in environments that are hostile, changeable and with physical and chemical properties at odds with the conditions found within constituent cells. To ensure normal cellular and organ function, organisms must generate and maintain a precise internal environment buffered from external conditions, with pH, osmolarity and ion concentration tightly regulated. The primary organ system that retains water and electrolytes and protects from the environment is the epidermis, which acts as an impermeable barrier elaborated with channels regulating passage of ions and small molecules including water [1]. Furthermore the epidermis in all organisms must also protect from outside-in assaults such as infectious agents, chemical insults, UV irradiation and physical damage [2].

An effective epidermal barrier is an ancient invention, and thus there are a number of features of the epidermis conserved across the vertebrate phylum [3]. The epidermis of adult vertebrates is stratified, composed of a basal (inner-most) stem cell layer residing on a basement membrane and overlaid by increasingly more mature superficial epidermal cell layers. Keratinocytes maintain adhesion through adherens junctions and desmosomes, mediated through the cadherin protein family, whilst impermeability of the tissue to small molecule diffusion is afforded by apically localised tight junctions in more superficial strata [2]. In addition to these features, terrestrial vertebrates require protection from dehydration. This is achieved through an additional lipid-rich superficial cornified layer, in which epidermal cells have undergone a process of terminal differentiation to become flattened, dead corneocytes, lacking organelles but containing heavily cross-linked insoluble protein structures and are enmeshed in an envelope of lipid lamellae. Desquamation leads to a continual loss of corneocytes superficially, with epidermal cell number maintained by replenishment from the basal stem cell layer [4]. In aquatic vertebrates such as fish there is no cornified layer and all cells of the epidermis remain metabolically active [1].

During mouse development, the basal compartment is established from the ectoderm as a monolayer during embryogenesis at E8.5, and then generates an overlying periderm layer [5]. This periderm constitutes the first front of the differentiating keratinocyte program. As development proceeds, this bilayered epidermis becomes multi-layered and individual strata are established. Eventually the periderm becomes the first cornified layer, is subsequently sloughed and replaced from intermediate cells of the underlying layer [6], [7]. In contrast to mouse, fish development occurs entirely externally and embryos are thus exposed to hypotonic conditions at early stages. To buffer against this, a cell lineage segregates prior to gastrulation, and generates a specialised epithelial layer, the Enveloping Layer (EVL) which constitutes a periderm monolayer encasing the entire embryo following gastrulation [8], and which contains membrane localised components of the tight junction complex [9]. A basal epidermal layer is established later during gastrulation. Similar to mouse and in contrast to the EVL, this basal layer is derived from the ventral non-neural ectoderm [10], and comes to reside under the EVL following gastrulation. Thus by somitogenesis stages, there is a bilayered epidermis covering the entire embryo, with each layer derived from different cell precursors.

The zebrafish epidermis remains bilayered until around 15 dpf (days post fertilisation) when intermediate layers become apparent [11]. The origin of these intermediate strata has not been demonstrated but is assumed to derive from the basal compartment. Whilst it has long been assumed that the EVL layer is lost during embryogenesis, mirroring the periderm of the mouse embryo, this has not been demonstrated [8]. Indeed mosaic cell labelling in zebrafish demonstrated that EVL cells persist until at least 9 dpf [12], however their fate beyond larval stages is unknown.

Analysis of murine epidermal development has been aided by a number of markers of different layers and stages of epidermal development. Much use has been made of the dynamic expression of keratins, which temporally and spatially reflect the differentiation status of epidermal cells. Keratin proteins form intermediate filaments within epithelial cells (cyto- or soft keratins), and also generate nail and hair structures decorating the epidermis (hard keratins) [13]. The human genome contains at least 54 keratin genes which encode proteins classified as either type I (acidic pI) or type II (basic pI) [14]. Together these form heterodimers which aggregate to generate the intermediate filaments of the cytoskeleton or of the hard cornifications of epidermal modifications [13]. While over half of keratin genes in the human genome are dedicated to the latter function, the numerous cytokeratins are expressed in specific domains of different epithelia. Simple epithelia of different organs express Keratin8 and 18, with certain simple epithelial tissues additionally labelled by other keratin pairs. Stratified epithelia express a distinct array of keratins, which again are specific to both tissue type and constituent strata. For example, Keratin5 and Keratin14 form a heterodimer specific to the basal cell layer of the epidermis, whilst the suprabasal layers are labelled by Keratin 1 and Keratin10 [13], [14]. Thus through in situ or antibody labelling, specific keratins have been used to characterise different epidermal layers, whilst cloning of the promoter regions of specific mammalian keratins has allowed misexpression of proteins in specific epidermal compartments.

Zebrafish contain much fewer keratin genes than mammals, reflecting the absence of hard epidermal modifications such as hair and nails. 16 type I and 7 type II keratins have been identified in the zebrafish genome [15], however a detailed survey of their expression in epithelia has not been conducted. It is likely that, as in mouse, they will show cell type specificities. Indeed the zebrafish *keratin4* gene has been shown to be expressed predominantly in the EVL [16] and the *keratin4* promoter has been used extensively to label the EVL from early stages [17]. To date a compact and robust promoter labelling basal epidermal cells is not available. A promoter from the gene encoding the transcription factor ΔNp63 has been used to label basal keratinocytes at 5 dpf but displays low and variable expression levels [18]. As the basal layer contains the epidermal stem cells, a strong and robust promoter to label these cells would be invaluable to researchers wishing to manipulate and study the epidermal stem cell niche in the zebrafish model system.

Here we demonstrate that the type I keratin encoding gene *krtt1c19e*, is broadly expressed in the basal epidermal cell layer, and identify an upstream promoter region which recapitulates this expression pattern in germline transgenics. Using this and the *krt4* promoter to drive an inducible form of Cre recombinase in the two cell layers of the embryonic epidermis, we trace the fate of the periderm in zebrafish and the origin of the external epidermal cell layer in adult zebrafish. We show for the first time that the EVL is transient and is shed gradually during metamorphic stages, being replaced from basally derived cells. Thus all strata of the adult zebrafish epidermis can be reconstituted from the basal compartment, highlighting that a system of epidermal renewal from a basal stem cell compartment likely existed prior to adaption to land.

## Materials and Methods

### Fish Husbandry and Cre Reporter Line

Zebrafish were housed in the IMCB Zebrafish facility and embryos used for analysis obtained through natural crosses and staged according to Kimmel et al. (1995) [19]. For lineage analysis, we used the *ubi:switch* reporter line *Tg(−3.5ubb:loxP-EGFP-loxP-mCherry)^cz1701^* [20]. All animal procedures in this study complied with the National Advisory Committee For Laboratory Animal Research (NACLAR) Guidelines set out by the Agri-Food and Veterinary Authority (AVA) of Singapore and was overseen by the Institutional Animal Care and Use Committee (IACUC) of the Biological Resource Centre BRC (IACUC Protocol Number: 090435).

### RNA Isolation and RT-PCR

RNA was isolated using Trizol (Invitrogen) from WT samples or water control, and cDNA generated using Superscript III Reverse Transcriptase (Invitrogen). Detection of *krtt1c19e* by PCR used the following forward and reverse oligos: F: CTCTTGAGAAAGCCAATGCTG; R: ACCTGTCCACTCATTTGATCG. *α-actin* was amplified as a positive control using these primers: F: TGGCATTGCTGACCGTATGC; R: GTCATGGACGCCCATTGTGA.

### Transgene Construction, Embryo Injection and Transgenesis

To generate transgene constructs, the promoters of both *krtt1c19e* and *krt4* [17] were cloned into a 5′ entry clone of the Tol2Kit [21], and all transgene constructs generated by Gateway recombination using LR Clonase II Plus enzyme (Invitrogen). Both the *krtt1c19e:Cre^ERt2^* and *krt4:Cre^ERt2^* constructs carried the *myl7:egfp* transgene in cis as a means to identify carriers. Plasmid DNA for injection was isolated using HiSpeed Plasmid Midi Kit (Qiagen). To generate transgenic zebrafish lines, 30 ng/µl plasmid DNA and 30 ng/µl t*ol2* RNA were co-injected into 1-cell stage embryos using a PLI-100 microinjector (Harvard Apparatus).

### In-situ Hybridisation and Immunostaining

The full 1.49 kb cDNA of *krtt1c19e* was cloned into pGEMT-Easy (Promega) and a full-length antisense in situ DIG-labelled RNA probe was generated using T7 RNA polymerase (Roche) following *Spe*I plasmid linearization. A shorter DIG-probe corresponding to only the final 651 bp (*krtt1c19e-3′)* was also made from this plasmid by linearizing in the middle of the cDNA with *Bgl*II prior to T7 polymerase transcription. A 5′ probe corresponding to the first 756 bp of *krtt1c19e (krtt1c19e-3′)* was generated by cloning a PCR-derived cDNA fragment into pGEMT-Easy, linearising with *Sal*I and transcribing with T7 RNA polymerase. Whole mount RNA in-situ hybridisation developed with NBT/BCIP was performed as described [22], whilst fluorescent in situ hybridisation was performed as per Brend and Holley (2009) [23], using fluorescein-conjugated tyramide. Following fluorescent in situ hybridisation, embryos were either immunostained for ΔNp63, or cryosectioned. Sectioning was performed using a Leica CM1900 cryostat at 16 µm thickness, and sections were then fluorescently immunostained. Immunostaining on sections or whole mount larvae was performed as following Asharani et al (2012) [24] using the following primary antibodies: chicken anti-eGFP (1∶500; #ab13970, Abcam), rabbit anti-DsRed/mCherry (1∶250; #632496, BD Biosciences), mouse anti- ΔNp63 (1∶100, sc-8431, Santa Cruz), mouse anti-ZO1 (1∶100; Invitrogen), mouse anti-E-Cadherin (1∶200; #610181; BD Bioscience) and rabbit anti-pan-Cadherin (1∶100, C3678, Sigma). Fluorescent conjugated secondary antibodies (1∶400; Jackson ImmunoResearch or Invitrogen) were used for detection of primary antibodies. Counterstaining of nuclei was performed using DAPI (1 µg/ml; Invitrogen) whilst Alexafluor-647-Phalloidin (33 ng/µl; Invitrogen) was used to visualise cell membrane associated cortical actin.

### Genetic Labelling through Tamoxifen Treatment and Transplantation

Permanent labelling of keratinocytes in *krt4:cre^Ert2^*; *ubi:switch* and *krtt1c19e:cre^Ert2^*; *ubi:switch* transgenics was achieved by pulsed activation of Cre^ERt2^ with 4-hydroxytamoxifen as per Lee et al, (2013) [25]. For each timecourse experiment, we imaged 8 fish at 4 different timepoints and repeated the experiement 3 times per genotype.

To generate clones of genetically labelled basal keratinocytes, deep cells from dome stage *krtt1c19e:lyn-tdtomato; krt4:lyn-egfp* double transgenic embryos were transplanted to age-matched wild-type hosts layer as described [26]. 5 individuals were imaged at 7 dpf and then assessed every 4 days for evidence of eGFP expression in EVL cells.

### Imaging

Confocal images were taken on a Zeiss LSM700 or an Olympus BX61 Fluoview microscope, whilst brightfield or Nomarski images were taken on a Zeiss AxioImager M2. Low-magnification fluorescent images of adult transgenic fish were taken on a Leica MZ16FA. In all experiments, multiple individuals or sections were examined and representative micrographs imaged.

To follow the expansion or loss of mCherry expressing cells upon permanent Cre-lox mediated labelling, floxed individuals were imaged every 4 days from 12 days onwards. Fish were anaesthetized with 0.02% Tricaine (buffered to pH 7.0) and mounted in 3% methyl cellulose. A post-anal region was iteratively re-imaged using a DM6000B Leica microscope. After imaging, the embryos were washed in facility water and returned to the fish facility.

## Results

### Identification of *krtt1c19e* as a Predominantly Basal Epidermal Marker

**Figure 1:**
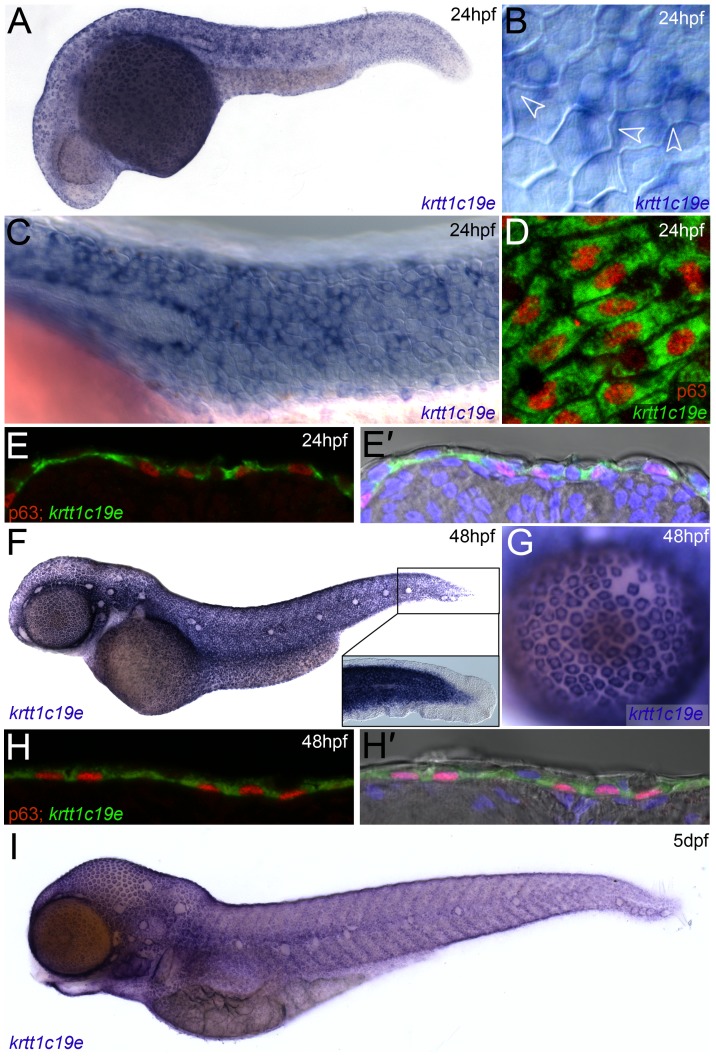
Expression of *krtt1c19e* in basal keratinocytes. In situ hybridisation of *krtt1c19e* at 24 hpf (A–E’), 48 hpf (F–H’) and 5 dpf (I), imaged laterally (A–D, F–G, I) or after cryosectioning (E–E’, H–H’). Overviews of embryos are shown at 24 hpf (A), 48 hpf (F) and 5 dpf (I), showing broad skin expression. Epidermal cells were visualised by counterstaining with DAPI (blue – E’, H’) or by Nomarski optics (B–C, F inset, E’, H’), and basal cell nuclei were immunolabelled using an antibody against ΔNp63 (red D–E’, H–H’). Strong *krtt1c19e* epidermal expression can be seen in the basal keratinocytes with the borders of overlying EVL cells intersecting basal cells (B - arrowheads). A gap in the *krtt1c19e* in situ signal is seen in the epidermis corresponding to the location of the migrating lateral line primordial (C). *krtt1c19e* expressing keratinocytes have ΔNp63 immunoreactive nuclei (D–E’, H–H’) and are seen below EVL cells in cryosections (E’, H’). A higher magnification of the tail region of the 48 hpf embryo is shown inset (F), with expression excluded from the fin epithelium, whilst a single layer of keratinocytes can be seen over the eye as part of the cornea (G).

To identify genes predominantly expressed in the basal epidermis, we exploited the lateral displacement of basal epidermal cells by the posterior lateral line primordium during its migration. This displacement generates a gap in the staining pattern of basal epidermal markers such as ΔNp63 (Figure S1A–A’), whilst overlying EVL cells are unaffected (see timelapse movies in [27]). We scanned an online database of wholemount in situ hybridisation patterns for epidermally expressed genes which show a gap in staining corresponding to the primordium. The type I keratin designated *krtt1c19e* [15] showed an appropriate pattern at 24 hpf [28], and the expression of this gene was further assessed. RT-PCR indicated expression at all stages of development up to adulthood, including the 2-cell stage (Figure S2A). Such early expression, indicative of maternal mRNA contribution, was confirmed by in situ hybridisation (data not shown). Using a full-length in situ probe, specific expression in the epidermis was first noted weakly by 15 hpf (Figure S2B, C), and was detected from 24 hpf up to at least 5 dpf (Figure 1A, 1F and 1I), with strong signal noted from 48 hpf onwards. We demonstrated expression was predominantly in basal epidermal cells and not EVL cells from 24 hpf onwards in a number of ways. Firstly, through Nomarski optics, we could identify unlabelled EVL cells overlying the labelled cells (Figure 1B; Figure S2E, S2G). Secondly, we could see labelled cells outline the primordium (Figure 1C; Figure S2F). Thirdly, we noted that the staining appeared to label only a single layer of the bilayered epidermis (for example of the cornea [29]; Figure 1G). Finally, immunostaining *krtt1c19e* fluorescent in situs with the basal keratinocyte nuclear marker, ΔNp63, showed consistent co-labelling of cells in a lateral view (24 hpf - Figure 1D). Cryosections of 24 and 48 hpf co-stained embryos indicated labelled cells occurred in a monolayer (Figure 1E–E’; 1H–H’), found below the (ΔNp63 and *krtt1c19e* negative) EVL layer, as visualised by DAPI counterstaining and Nomarski optics. Curiously we observed that the larval fin fold epithelium does not express *krtt1c19e* at any stage (Figure 1F; Figure S2H), despite the presence of basal keratinocytes here. We also noted a small number of EVL cells were faintly stained using the more sensitive fluorescent in situ hybridisation (Figure S2D), suggesting that some EVL cells do express very low levels of *krtt1c19e* or a transcript with some sequence homology. In situ probes against either the first or second half of the *krtt1c19e* cDNA show an identical pattern, albeit weaker than the full length (Figure S3A–F).

### Isolation of a *krtt1c19e* Promoter and Generation of Germline Transgenics

**Figure 2:**
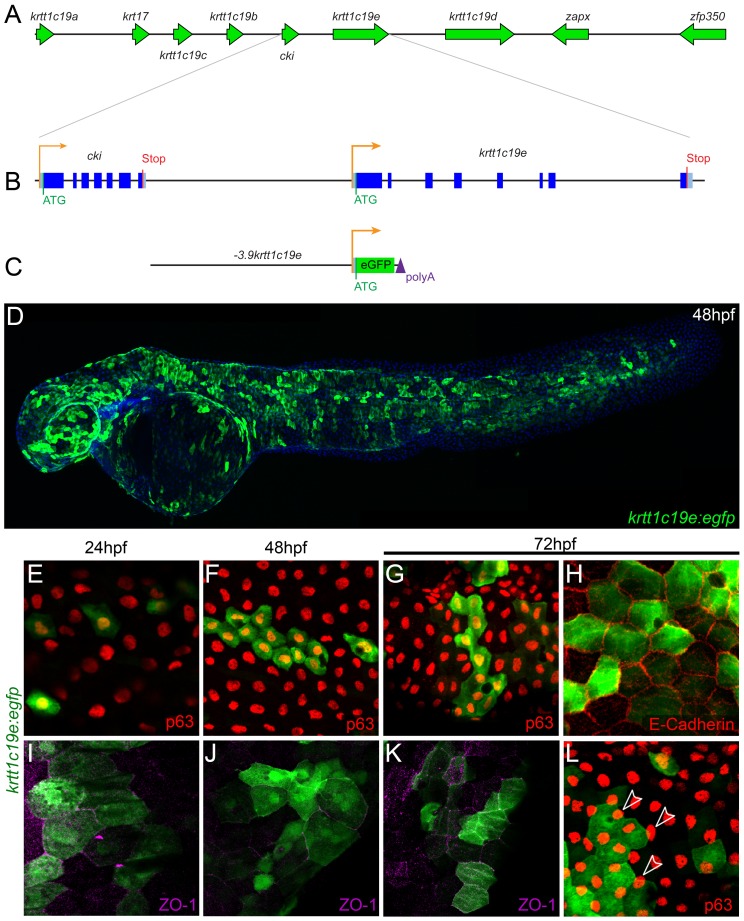
Isolation and transient activity of a *krtt1c19e* promoter. Map of the genomic context of the *krtt1c19e* gene in the type I keratin cluster on chromosome 19 (A) with a schematic of the intron/exon structure of the *krtt1c19e* gene and the upstream *cki* gene (B). The entire sequence upstream of *krtt1c19e* to the end of the *cki* was isolated and cloned upstream of *egfp* (C). Injection of this *krtt1c19e:egfp* construct into embryos yielded limited epidermal expression at 24 hpf but widespread eGFP expression by 48 hpf (C). **E–L:** Micrographs of eGFP positive epidermal cells in both the basal layer (E–H) and EVL (I–L), demonstrated by co-immunofluorescent labelling with antibodies against eGFP (green – E–L), ΔNp63 (red – E–G, L), E-cadherin (red - H) and ZO-1 (purple – I-K). At 24 hpf (E, I), 48 hpf (F, J) and 72 hpf (G–H, K–L) both ΔNp63 and E-cadherin positive basal cells are eGFP positive as are ZO-1 positive EVL cells. eGFP positive EVL cells can be seen above ΔNp63 positive nuclei (arrowheads - L).

Given that *krtt1c19e* is highly and predominantly expressed in basal keratinocytes, we sought to isolate its regulatory regions as a means of driving exogenous genes in this cell layer. This gene sits within a type I keratin gene cluster on chromosome 19 (Figure 2A), with the start of transcription only 3.9 kb downstream from the end of the preceding gene (*cki* – Figure 2B). This suggested *krtt1c19e* might possess a relatively small promoter. We cloned 3.9 kb upstream from the start of translation of the *krtt1c19e* gene into a 5′ gateway entry vector of the Tol2kit [21], and recombined it upstream of eGFP (Figure 2C). Transient analysis upon injection of this construct into embryos showed limited epidermal expression at 24 hpf, which became extremely widespread from 48 hpf onwards (Figure 2D). To ascertain if the cells labelled were indeed basal cells as expected, we counterstained injected embryos with basal and EVL specific markers. From 24 hpf to 72 hpf, embryos contained eGFP cells which were ΔNp63 positive (Figure 2E–G), and were outlined with strong E-cadherin staining at 72 hpf (Figure 2H), both indicative of basal keratinocyte expression. At all stages however, we also noted GFP labelling of overlying ZO-1 positive EVL cells (Figure 2I–K), confirmed by eGFP cell borders bisecting underlying ΔNp63 nuclei (Figure 2L). Thus the *krtt1c19e* promoter can transiently drive gene expression in both cell layers of the embryonic and larval epidermis.

**Figure 3:**
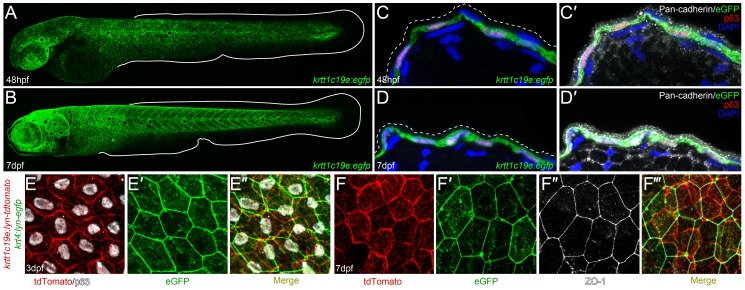
Characterization of *krtt1c19e :egfp* transgenic larvae. **A–D’:** Confocal images of eGFP expression in germline *krtt1c19e:egfp* transgenic larvae at 48 hpf (A, C–C’) and 7 dpf (B, D–D’). **A–B:** Lateral overviews of transgenic larvae indicating the *krtt1c19e* promoter drives eGFP expression in the epidermis of zebrafish larvae at all locations except the fins (extent of medial fins outlined by white line). **C–D’:** Immunofluorescent labelling of transgenic larvae cryosections demonstrates co-expression of eGFP (green; C–D’) and ΔNp63 (red; C–D’) in basal keratinocytes. Counterstaining with DAPI (blue; C–D’) and Pan-cadherin (white; C’, D’) highlights the eGFP negative region overlying EVL (demarcated by dashed lines; C’, D’). **E–F’’’:** Confocal images of the epidermis of germline *krtt1c19e:lyn-tdtomato; krt4:lyn-egfp* double transgenic larvae at 72 hpf (E–E’’) and 7 dpf (F–F’’’) immunofluorescently stained for eGFP (green; E’–E’’, F’, F’’’), tdTomato (red; E, E’’, F, F’’’), ΔNp63 (white; E, E’’) and ZO-1 (white; F’’–F’’’). The expression of membrane bound tdTomato delineates the ΔNp63 positive basal keratinocytes from the eGFP expressing ZO-1 positive EVL cells.

Due to the variable transgene copy number partitioned to cells upon transient injection, promoters often show activity in ectopic locations, not representative of their true activity domains. To test this, we generated germline transgenics from the injected embryos and identified a number of founder lines, the majority of which showed identical expression patterns. We could first detect eGFP weakly at 36 hpf in the epidermis, but by 48 hpf expression is robustly detected in keratinocytes over the head and body of the fish, but excluded from the fin fold epithelium (Figure 3A), mirroring the endogenous *krtt1c19e* mRNA. We observed similar expression pattern at all larval stages to 7 dpf (Figure 3B). The promoter drove eGFP expression strongly and predominantly in the basal ΔNp63 positive keratinocytes at both 48 hpf to 7 dpf with the EVL unlabelled, as demonstrated by immunofluorescent staining of cryosections (Figure 3C–D’). To better visualise basal keratinocyte morphology, we used this promoter to label the cell membrane with lyn-tdTomato. The lyn tag acts as a myristoylation and palmitoylation substrate, and once modified will direct proteins to the cell membrane [30]. We crossed the resulting transgenic line with another transgenic line expressing membrane tethered eGFP in the EVL using the *krt4* promoter. At both early (Figure 3E–E’’; Figure S4A–A’’’) and late (Figure 3F–F’’’; Figure S4B–B’’’) larval stages, the two membrane labels were non-overlapping. The ΔNp63 basal keratinocytes were labelled by the membrane tethered td-Tomato and distinct from the overlying ZO-1 positive EVL cells which are demarcated by the lyn-eGFP. This further highlighted the specific basal expression of the *krtt1c19e* promoter.

**Figure 4:**
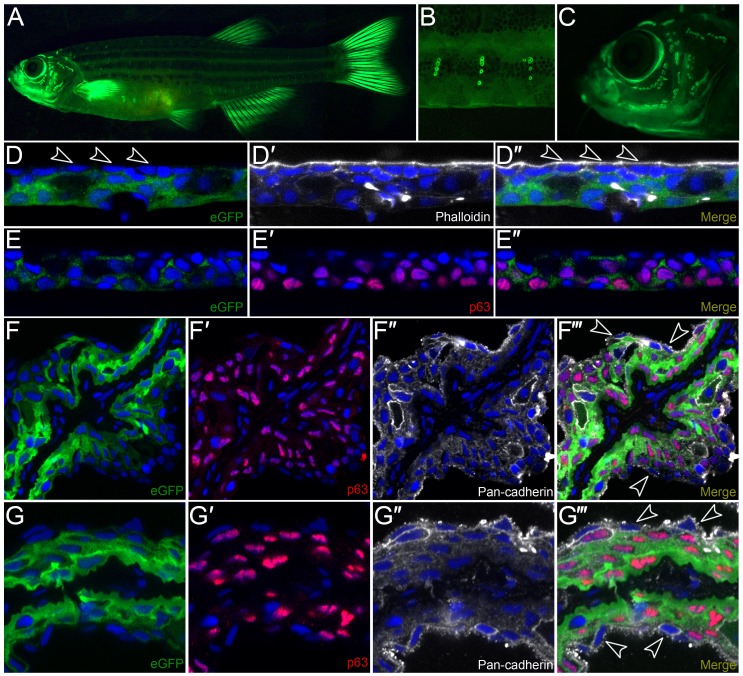
Characterization of *krtt1c19e:egfp* transgenic adult zebrafish. **A–C:** Lateral micrographs of *krtt1c19e:egfp* transgenic adult zebrafish, showing eGFP expression weakly in the trunk region but strongly in the fins (A). Expression is also seen associated with neuromasts of the lateral line system in the body (A, B) and head (C). **D–G’’’:** Immunostaining of cryosections from the trunk region (D–E’’) or fin (F–G’’’) of transgenic adults demonstrates expression is in basal and suprabasal epidermal cells, with no expression visible in the superficial epidermal stratum (arrowheads D–D’’). eGFP is shown in green (D, D’’, E, E’’, F, F’’’, G, G’’’) and co-localises with ΔNp63 (red; E’–E’’, F’, F’’’, G’, G’’’) in basal and suprabasal keratinocytes, and is excluded from the most superficial keratinocytes (e.g. arrowheads F’’’, G’’’) visualised by DAPI (blue; D–G’’’), Phalloidin staining (white, D’–D’’) or Pan-cadherin staining (white; F’’–F’’’, G’’–G’’’).

We raised the *krtt1c19e:egfp* transgenic larvae to adulthood, and noted GFP expression is sustained, albeit faintly, in the trunk epidermis (Figure 4A), whilst there was now strong GFP signal associated with the neuromasts of both the body and head lateral line systems (Figure 4B–C). Co-staining of *krtt1c19e:lyn-tdtomato* adults with antibodies against p63 and tdTtomato indicated that this labelling was restricted to p63 positive epidermal cells surrounding the neuromast (Figure S5A–B”). In contrast to the larval expression pattern, there was strong epidermal expression in all fins (Figure 4A). Immunostaining of cryosectioned transgenics was used to determine which epidermal cell layers were GFP positive. Counterstaining with DAPI, ΔNp63, Phalloidin and Pan-cadherin demonstrated that the promoter labels the basal and suprabasal layers of the epidermis, but does not label the most superficial layer of the epidermis at trunk or fin locations (Figure 4D–G’’’). This indicates that the *krtt1c19e* promoter can drive expression in basal (or suprabasal) epidermal layers at all stages, but is inactive in the superficial EVL.

### The EVL is a Transient Structure and is Replaced during Metamorphosis

**Figure 5:**
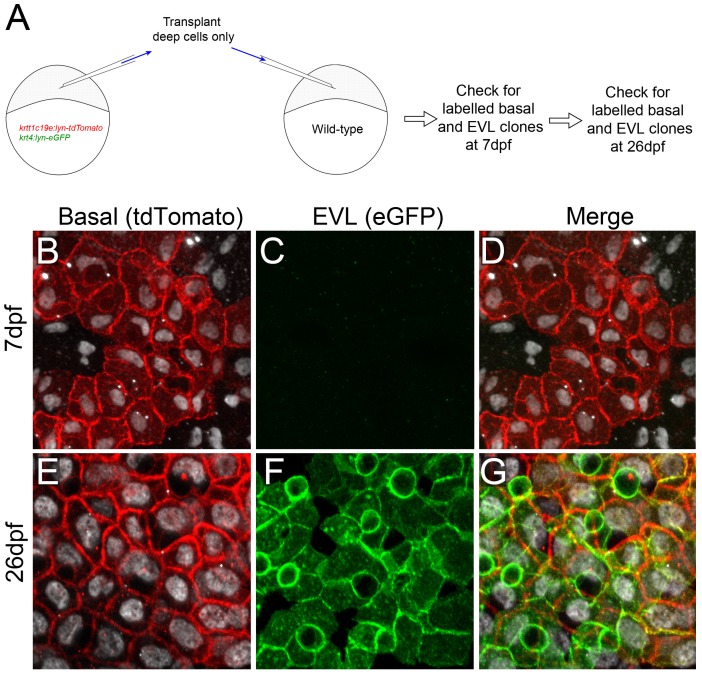
Transplantation of basal epidermal cells demonstrates contribution to the EVL at late stages. **A:** Schematic of transplantation strategy to test if the EVL of post metamorphosis larvae derives from the basal layer. Deep cells from *krtt1c19e:lyn-tdtomato; krt4:lyn-egfp* double transgenic embryos were transplanted into wild-type embryos after segregation of the EVL lineage at 4.3 hpf. Labelling of different epidermal cells was checked at 2 timepoints. **B–G:** Confocal images of the epidermis of representative recipient embryo at 7 dpf (B–D) and 26 dpf (E–G), immunostained for ΔNp63 (white; B,D–E, G), tdTomato (red; B,D–E, G) and eGFP (green; C–D, F–G). Transplanted deep cells only generate clones of basal cells expressing lyn-tdTomato at 7 dpf (B–D), but do not contribute to the EVL. The superficial layer is eGFP positive at 26 dpf indicating it is descended from the basal cells (E–G). n = 5 transplants were followed.

Having a means to label the basal keratinocytes prompted us to test if these cells eventually generate the entire epidermis as in mammals, or if the EVL is retained throughout the life of the fish. We used two methods to test if the superficial EVL is derived from the basal cells. Firstly we employed a transplantation approach to generate clones of basal cells carrying transgenes labelling the basal keratinocytes and EVL cells. To do this, we used the *krtt1c19e:lyn-tdtomato; krt4:lyn-egfp* double transgenic fish described above and the fact that the EVL lineage restriction to superficial periderm cells occurs prior to 4 hpf. At this time point deep cells of the blastula are unable to give rise to periderm cells of the larva [8]. We thus transplanted deep cells from double transgenic embryos into unlabelled hosts at dome stage (4.3 hpf) (see Figure 5A for experimental outline). We confirmed at embryonic and larval stages that whilst the hosts contained a mosaically labelled basal epidermal layer (Figure 5B, D), there had been no transplantation of EVL cells, as all cells in this layer were unlabelled (therefore host derived – Figure 5C, D). Daughter cells of these basal cells will share the same genotype including the *krt4:lyn-egfp* transgene, and thus EVL cells will be marked by lyn-eGFP if, and only if, they are derived from basal cells. By 26 dpf we could observe expression of lyn-eGFP in the superficial layer of the epidermis (Figure 5F). In all cases, eGFP positive EVL clones were found above tdTomato labelled basal cells (Figure 5E, G). Together this strongly suggests that at least a proportion of the EVL is replaced by basal keratinocytes and that the embryonic EVL is transient.

**Figure 6:**
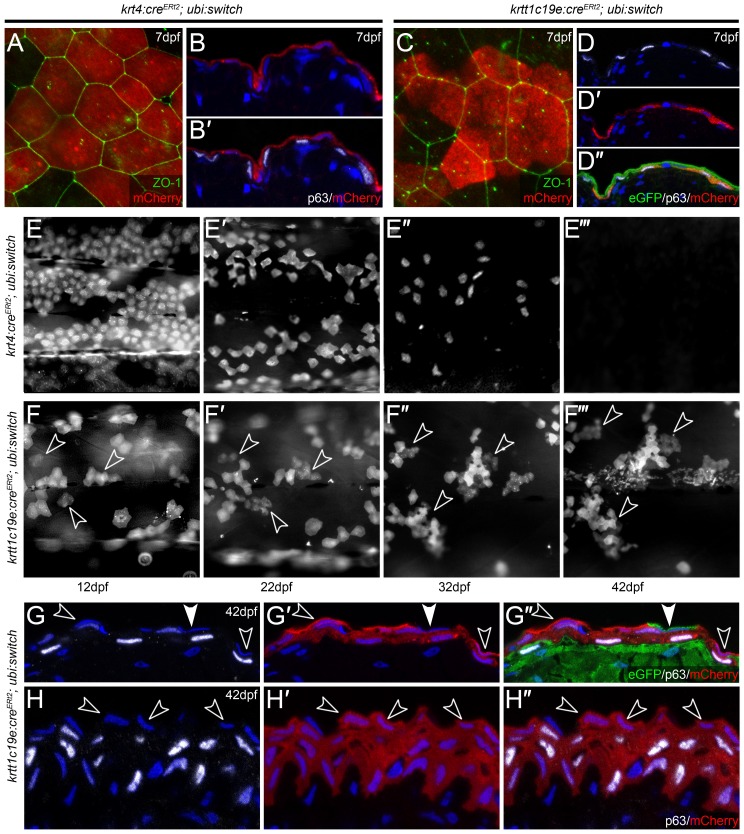
Embryonic EVL is lost and is replaced by cells from the basal epidermis during metamorphosis. **A–D’’:** Confocal images of lateral views (A, C) and transverse cryosections (B–B’, D–D’’) of *krt4:Cre^ERt2^; ubi:swtch* (A–B’) and *krtt1c19e:Cre^ERt2^; ubi:swtch* (C–D’’) at 5 dpf following 4-OHT mediated Cre conversion, and which have been immunofluorescently labelled with antibodies against mCherry (red; A–C, D’–D’’), ZO-1 (green; A, C), ΔNp63 (white; B’, D, D’’) and eGFP (green; D’’) and counterstained with DAPI (blue; B–B’, D–D’’). Upon treatment of 4-OHT, *krt4:Cre^ERt2^* drove recombination, and thus mCherry expression, in ZO-1-positive/ΔNp63-negative EVL cells (A–B’), whilst *krtt1c19e:Cre^ERt2^* induced recombination in ΔNp63-positive/ZO-1-negative basal keratinocytes (C–D’’). **E–F’’’:** Time course of floxed *krt4:Cre^ERt2^; ubi:swtch* (E–E’’’) and *krtt1c19e:Cre^ERt2^; ubi:swtch* (F–F’’’) showing the same region of mCherry positive cells on the flank of representative individuals. Fluorescent images were taken at 12 dpf (E, F), 22 dpf (E’, F’) 32 dpf (E’’, F’’) and 42 dpf (E’’’, F’’’), and show that EVL cells are gradually lost, whilst clones of basal keratinocytes expand and stratify. n = 24 per genotype. **G–H’’:** Transverse cryosections of the trunk (G–G’’) and fin (H–H’’) epidermis of 42 hpf floxed *krtt1c19e:Cre^ERt2^; ubi:switch* transgenics, immunostained with antibodies against ΔNp63 (white; G, G’’, H, H’’), mCherry (red; G’–G’’, H’–H’’) and eGFP (green, G’’). Nuclei of all cells in the epidermis are marked by DAPI staining (blue; G–H’’). In contrast to 7 dpf (C–D’’), mCherry is now found in both suprabasal and the most superficial ΔNp63-negative cell layers (examples of the latter highlighted by open arrowheads). Occasional superficial cells, not derived from floxed basal cells, can be seen (green cell highlighted by closed arrowhead G–G’’).

To confirm this conclusion, we used the Cre-lox system to permanently label both basal keratinocytes and EVL. We generated *krtt1c19e:cre^Ert2^* and *krt4:cre^Ert2^* transgenic lines and crossed these to the *ubi:switch* Lox reporter line [20]. Upon activation of Cre recombinase, this reporter switches from expression of eGFP to mCherry. We treated *krt4:cre^Ert2^*; *ubi:switch* larvae with 4-Hydroxytamoxifen (4-OHT) from 30 to 78 hpf to activate Cre recombinase mediated switching. This labelled the EVL specifically with mCherry at larval stages (Figure 6A–B’). Similarly treatment of *krtt1c19e:cre^Ert2^*; *ubi:switch* larvae with 4-OHT for 90 minutes at 72 hpf led to robust labelling of the basal epidermis when visualised at 7 dpf (Figure 6C–D’’). We followed larvae over the following 2 months and noted that EVL cells labelled at early larval stages do persist until approximately 1 month, but found that they are gradually lost from the epidermis at metamorphosis stages. Clones thinned out from 15 dpf onwards such that at 1 month only a handful of cells remain, and by 42 dpf, we could not observe labelled cells in the majority of individuals (Figure 6E–E’’’). This indicated that the vast majority (and likely, all) EVL cells are not permanent. To confirm that the superficial cells are being replaced from basal keratinocytes, we followed clones of mCherry positive cells from the floxed *krtt1c19e:cre^Ert2^*; *ubi:switch* transgenics. In contrast to EVL labelled clones, we noted both expansion and stratification of basal clones (Figure 6F–F’’’). To demonstrate that these cells expand to contribute to the most superficial stratum, we sectioned through mCherry labelled clones of *krtt1c19e:cre^Ert2^*; *ubi:switch* double transgenics at 42 dpf and immunostained for mCherry, eGFP and ΔNp63. We observed that within both the trunk (Figure 6G–G’’) and fin (Figure 6H–H’’) epidermis, basal cells clones contribute to all layers of the epidermis, including the most superficial cells (as marked by DAPI), suggesting that not only is the EVL lost, but it can be replaced from basal cells. Note that each clone also contains EVL cells which are not floxed, expressing eGFP rather than mCherry (example shown in Figure 6G). Due to the mosaic nature of the Cre mediated recombination, it is not possible to determine if such cells represent perduring embryonic EVL cells or are derived from neighbouring unfloxed basal keratinocytes. Nonetheless our *krt4:cre^Ert2^*; *ubi:switch* data conclusively shows that the vast majority of embryonic EVL cells are lost within the first month after fertilization.

## Discussion

There is an increasing use of the zebrafish system in the epidermal field, underscored by a growing awareness of the similarities between mammalian and piscine epidermis at the genetic, cellular and organ levels. Being aquatic, fish have no need for a lipid rich water-impermeable stratum corneum, which evolved after the transition to land [3], and this lack of cornification represents the major difference between mouse and fish epidermis. However we propose that the fish presents an excellent system to study the biology of basal/suprabasal keratinocytes and the dermis, in isolation from secondary cornification defects. Indeed ultrastructural and marker analysis [11], [31] identified many anatomical and cytological similarities between fish and mammalian skin, including stratification, adhesion structures and dermal stroma content. Furthermore, genetic analyses, driven in part by genetic screens [32], [33], have led to the growing awareness that the genetic basis of zebrafish epidermal development shares much with mouse and human genetics, and can model human disease. Attenuated function of *kindlin-1* [34], *integrinα3*, *lamininα5* [35], *irf6* [36], *epcam* [37], *lgl2* [31], *ikk1* [12], *col17a1* [38] and *spint1a* [39] all generate defects in the zebrafish epidermis or periderm which are comparable to loss in mammalian systems, underscoring conservation of gene function. Indeed in some cases the mutants model the human disease accurately. For example, mutations in the zebrafish orthologues of human *FRAS1, FREM1* and *FREM2* recapitulate the epidermal blistering of distal appendages seen in Fraser Syndrome [35]. Importantly, the basal epidermal transcription factor ΔNp63, which has a mitogenic role in mammalian basal keratinocytes, has an identical function in zebrafish. Loss of zebrafish ΔNp63 reduced basal keratinocyte proliferation and lead to blistering and epidermal lysis [10], [40], and shows strong phenotypic parallels with the human ectodermal dysplasia, ectrodactyly, and cleft palate syndrome [41]. Thus a basal epidermal stem cell population, and the genetic programs deployed therein, were an ancient invention in the vertebrate lineage.

In addition to the basal layer, the epidermis of zebrafish embryos contains a periderm layer, which is derived from the first cell lineage to segregate during development – the enveloping layer. Previously assumed to be lost during development, it had been shown that the EVL is retained until at least late larval stages [12], thus making its final fate unclear. It was possible that the lineage was retained throughout life and EVL cells were replaced by cell division within the stratum as seen in the epidermis of Drosophila [42]. We have shown for the first time using a Cre/Lox approach that, as in the mouse, the EVL/periderm is gradually lost in zebrafish albeit at a relatively late developmental stage. Both transplantation and employment of the Cre/Lox system demonstrated that the cells of the most superficial stratum are reconstituted from basal cells. These replacement superficial cells, derived from an alternate lineage, display all the hallmarks of the original EVL cells, namely labelling by the *krt4:lyn-egfp* transgene and presence of apical microridges. Thus we have shown that keratinocytes of all strata of the adult epidermis derive from the basal keratinocytes, and a basal keratinocyte layer able to contribute daughter cells to all epidermal strata likely evolved before the transition of vertebrates to land.

We noted a number of secretory cells in the epidermis are unlabelled in the floxed *krtt1c19e:cre^Ert2^*; *ubi:switch* transgenics, however this was expected as they segregate very early from epidermal progenitors within the non-neural ectoderm, prior to our Cre labelling [43], [44], [45]. Whilst the loss of the original EVL cells at metamorphosis was gradual we have not yet quantified the rate of turnover of the adult epidermis. Experiments are currently underway using later pulses of 4-OHT to measure this. It would be of further interest to employ the reagents we have presented here to assess the behaviour of different lineages during wound healing and assess if there is an increased turnover of cells in different strata. Indeed preliminary characterisation of the wound healing process in adult zebrafish shows extremely rapid re-epithelialisation [46]. Our reagents now permit dissection of the strata driving barrier re-establishment.

Finally, we have identified that *krtt1c19e* is strongly and predominantly expressed in basal epidermal cells. We observed some unexpected regionalisation of the epidermis through our in situ analysis, with *krtt1c19e* expression excluded from the larval fins, despite the presence of p63 positive basal epidermal cells in this location. Such a sub-regionalisation had not been described before and we are unsure of its significance. It was confirmed by use of the *krtt1c19e* promoter, which curiously showed a converse expression pattern in the adult with basal epidermal cells of the fins and surrounding neuromasts strongly labelled, whilst trunk epidermal cells weakly labelled. We surmise the promoter might be responding to an inhibitory signal from the larval fin apex, and is de-repressed in the adult fin and neuromast region. However it occurs, it demonstrates that the zebrafish epidermis shows regionalisation, mirroring the regionalisation of mammalian epidermis, where areas such as the palms are labelled by specific keratins [13].

It is impossible to assign homology of the *krtt1c19e* gene to a mammalian orthologue, as most zebrafish keratin proteins are more closely related to each other than any are to mammalian homologues. This precludes definitive orthology assignment based on phylogenetics, and may be due to an origin from tandem duplications or through intra-specific sequence convergence [15]. Nonetheless, the *krtt1c19e* promoter provides a useful means to drive strong expression in basal keratinocytes, allowing and precise mis-expression of genes in this stem cell type. Whilst this promoter presents a useful reagent, we suspect it may not be complete as there is a delay in observable eGFP levels compared to *krtt1c19e* in situ signals, and fortunately we do not observe maternal contribution of *egfp* which would be expected for a complete promoter. Nonetheless, it will assist imaging of basal keratinocyte morphology in real-time in a vertebrate model. The tools we present will assist the growing number of researchers using the zebrafish system to study epidermal development.

## Supporting Information

Figure S1 **Lateral line primordium migration displaces basal epidermal cells.** Confocal image of the lateral epidermis of a 28 hpf embryo immunofluorescently stained with an antibody against ΔNp63 (red; A–A’) and counterstained with DAPI (blue; A’). A gap in the basal epidermis is seen through displacement of the nuclei, and corresponds to the migrating primordium as indicated by the dense cluster of cell nuclei (A’).
(TIF)

Figure S2 **Timing of expression of** ***krtt1c19e*** **in the epidermis. A:** RT-PCR of *krtt1c19e* (upper gel) at stages given and compared to *β-actin* positive control (lower gel) showing expression of *krtt1c19e* at all stages. Negative water control is given in far right lane. **B–H:** In situ hybridisations of *krtt1c19e* detected fluorescently (D) or by chromogenic precipitate (B–C, E–H) at 15 pf (B–C), 24 hpf (D), 48 hpf (E–F) and 4 dpf (G–H). Lateral (B) and dorsal (C) view of *krtt1c19e* in situ hybridisation at 15 hpf, when specific epidermal expression can be discerned. Counter-staining fluorescent in situ hybridisations with an antibody against ΔNp63 (red; D) demonstrates that in addition to the predominant basal keratinocyte expression, there is some low level expression of *krtt1c19e* in the EVL (outlined in blue) at 24 hpf. The predominant expression of *krtt1c19e* in basal layers at 48 hpf and 4 dpf is demonstrated through imaging the boundary of overlying EVL cells with Nomarski optics (arrowheads; E, G) and observing the lateral displacement of *krtt1c19e* expressing cells by the primordium at the end of its migration (F). The expression of *krtt1c19e* remains excluded from the epidermis of the medial fin at 4 dpf (H).
(TIF)

Figure S3 **Independent in situ probes confirm** ***krtt1c19e*** **expression.** Micrographs of 24hpf (A, B), 48hpf (C, D) and 5 dpf (E, F) embryos hybridised with 5′ *krtt1c19e* (A, C, E) and 3′ *krtt1c19e* (B, D, F) in situ probes. Whilst sensitivity was reduced, in particular at 24hpf, expression in the epidermis was identical to that seen with the full length probe.
(TIF)

Figure S4 **Mutually exclusive expression of lyn-tdTomato and lyn-eGFP in the basal layer and EVL in** ***krtt1c19e:lyn-tdtomato; krt4:lyn-egfp*** **double transgenics. A–B’’’:** Confocal images of the epidermis of *krtt1c19e:lyn-tdtomato; krt4:lyn-egfp* double transgenic larvae at 3 dpf (A–A’’’) and 7 dpf (B–B’’’) immunofluorescently stained for eGFP (green; A’, A’’’, B’, B’’’), tdTomato (red; A, A’’’, B, F’’’), ZO-1 (white; A’’–A’’’) and ΔNp63 (white; B’’, B’’’). The *krtt1c19e* promoter drives expression in the ΔNp63 positive basal layer.
(TIF)

Figure S5 **Strong expression of the** ***krtt1c19e*** **promoter in epidermal cells surrounding the adult neuromasts.** Low (A–A”) and high (B–B”) magnification confocal images of cells surrounding trunk neuromasts of a *krtt1c19e:lyn-tdtomato* transgenic adult, immunofluorescently stained for tdTomato (red; A, B, A”, B”) and ΔNp63 (white; A’, B’, A”, B”). High level promoter activity is evident in p63 positive epidermal cells surrounding the neuromasts.
(TIF)
