# Supplementary figures and images for: Basal Keratinocytes Contribute to All Strata of the Adult Zebrafish Epidermis
Source: PLoS One. 2014 Jan 6;9(1):e84858. doi: 10.1371/journal.pone.0084858 (PMC3882266; doi:10.1371/journal.pone.0084858)

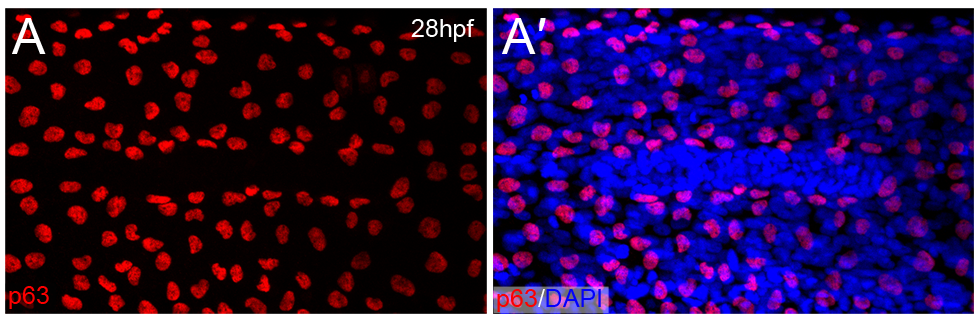

Supplement: Figure S1 — Lateral line primordium migration displaces basal epidermal cells. Confocal image of the lateral epidermis of a 28 hpf embryo immunofluorescently stained with an antibody against ΔNp63 (red; A–A’) and counterstained with DAPI (blue; A’). A gap in the basal epidermis is seen through displacement of the nuclei, and corresponds to the migrating primordium as indicated by the dense cluster of cell nuclei (A’). (TIF) [file pone.0084858.s001.tif]

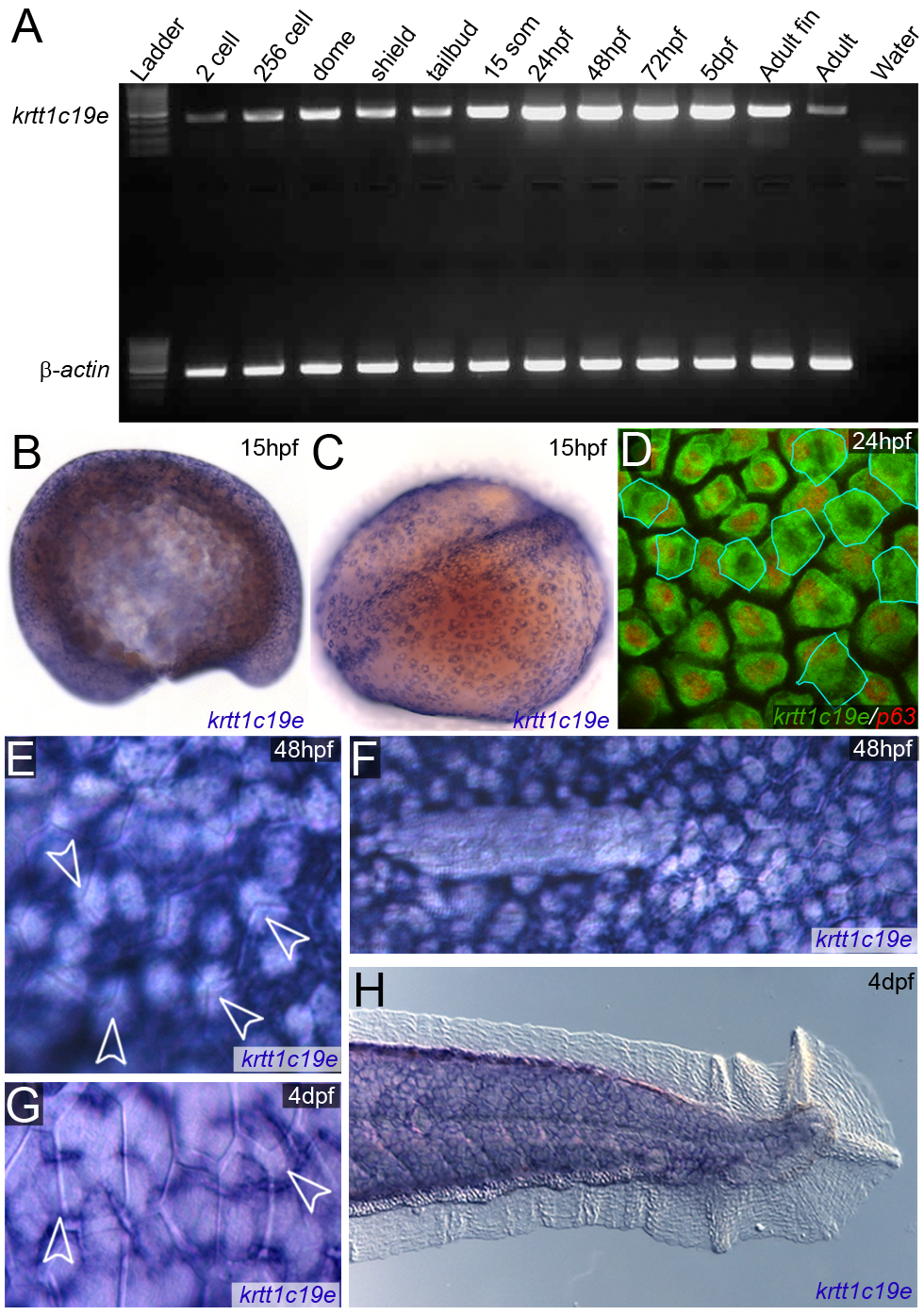

Supplement: Figure S2 — Timing of expression of krtt1c19e in the epidermis. A: RT-PCR of krtt1c19e (upper gel) at stages given and compared to β-actin positive control (lower gel) showing expression of krtt1c19e at all stages. Negative water control is given in far right lane. B–H: In situ hybridisations of krtt1c19e detected fluorescently (D) or by chromogenic precipitate (B–C, E–H) at 15 pf (B–C), 24 hpf (D), 48 hpf (E–F) and 4 dpf (G–H). Lateral (B) and dorsal (C) view of krtt1c19e in situ hybridisation at 15 hpf, when specific epidermal expression can be discerned. Counter-staining fluorescent in situ hybridisations with an antibody against ΔNp63 (red; D) demonstrates that in addition to the predominant basal keratinocyte expression, there is some low level expression of krtt1c19e in the EVL (outlined in blue) at 24 hpf. The predominant expression of krtt1c19e in basal layers at 48 hpf and 4 dpf is demonstrated through imaging the boundary of overlying EVL cells with Nomarski optics (arrowheads; E, G) and observing the lateral displacement of krtt1c19e expressing cells by the primordium at the end of its migration (F). The expression of krtt1c19e remains excluded from the epidermis of the medial fin at 4 dpf (H). (TIF) [file pone.0084858.s002.tif]

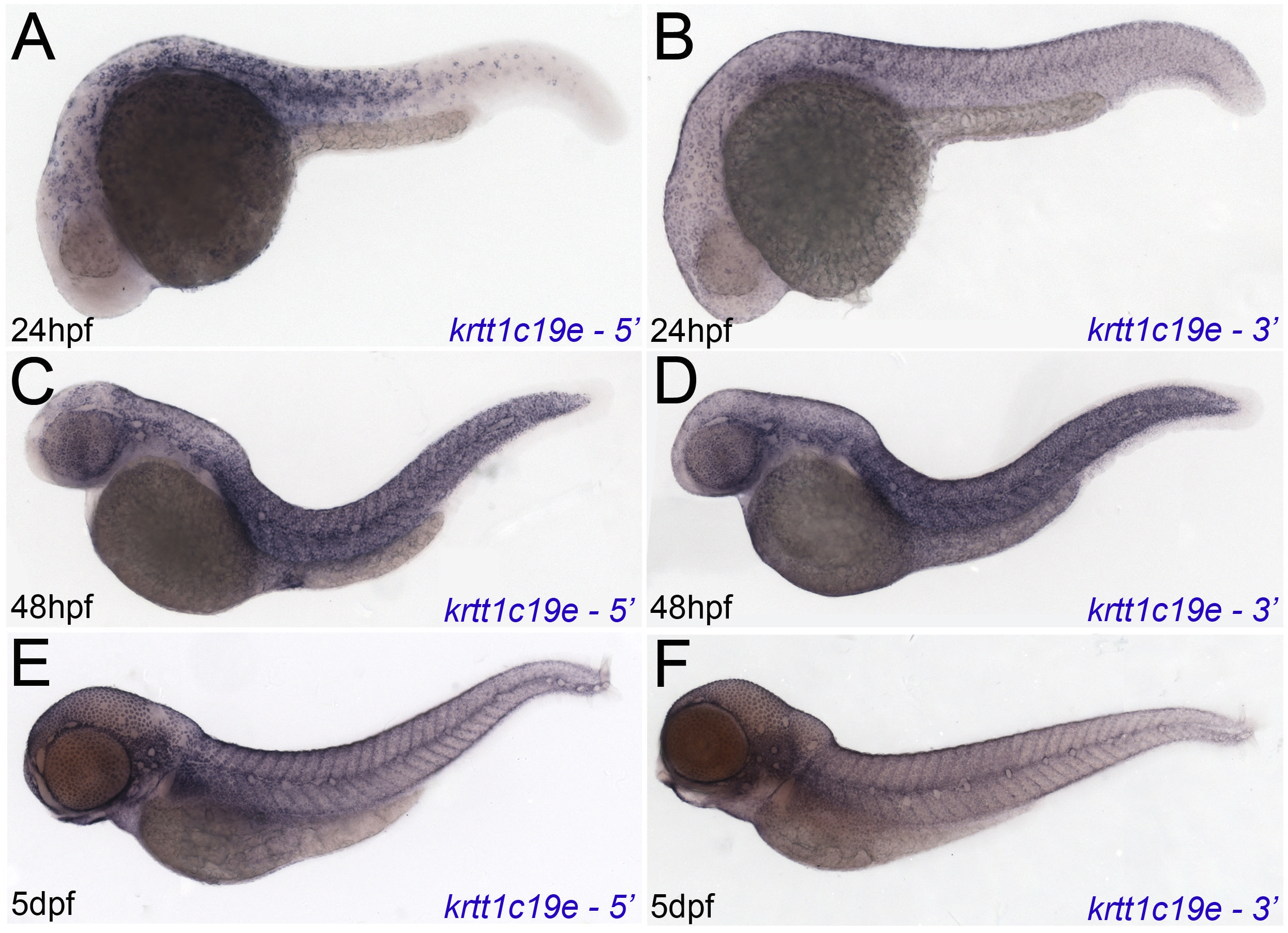

Supplement: Figure S3 — Independent in situ probes confirm krtt1c19e expression. Micrographs of 24hpf (A, B), 48hpf (C, D) and 5 dpf (E, F) embryos hybridised with 5′ krtt1c19e (A, C, E) and 3′ krtt1c19e (B, D, F) in situ probes. Whilst sensitivity was reduced, in particular at 24hpf, expression in the epidermis was identical to that seen with the full length probe. (TIF) [file pone.0084858.s003.tif]

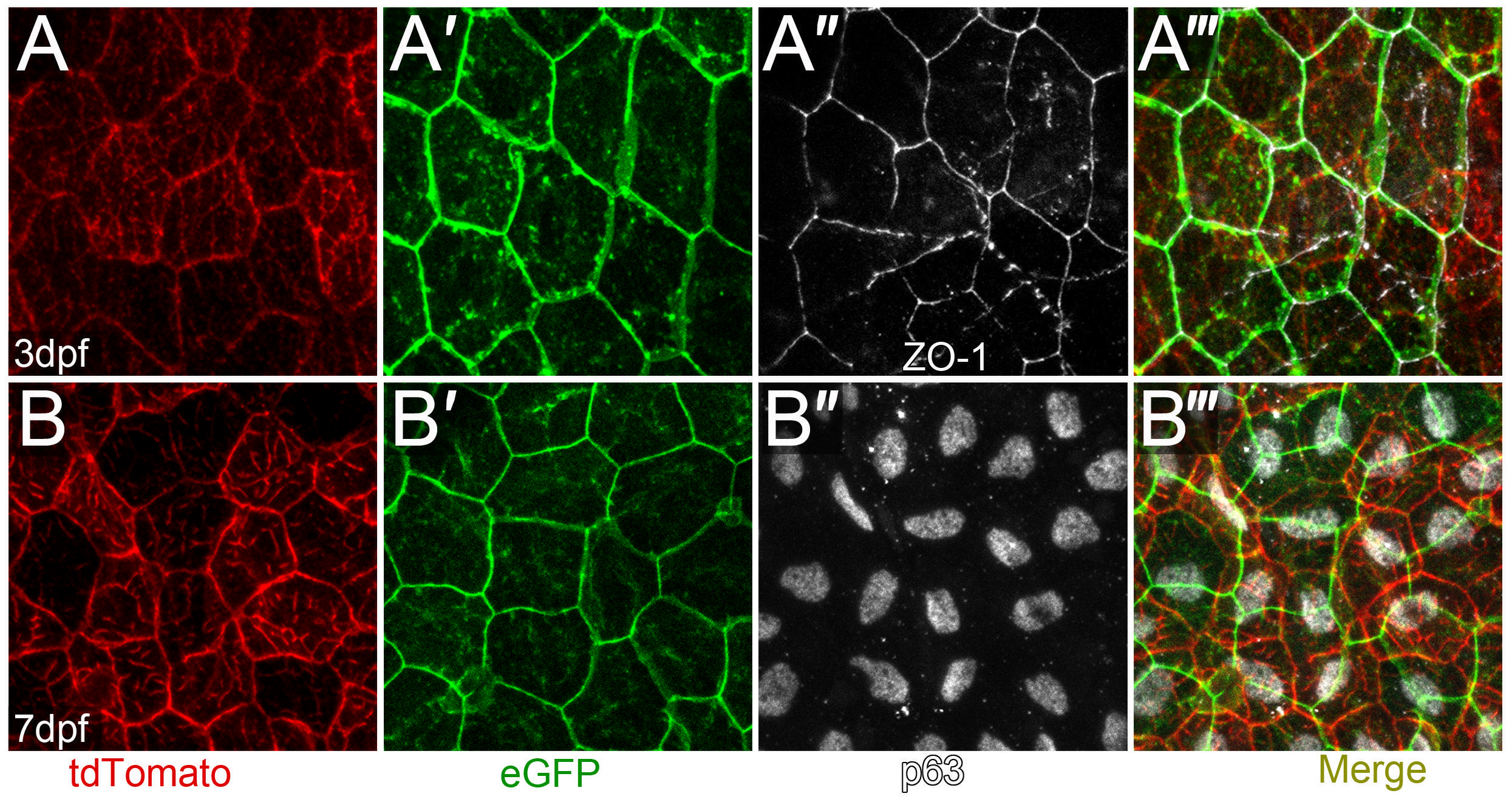

Supplement: Figure S4 — Mutually exclusive expression of lyn-tdTomato and lyn-eGFP in the basal layer and EVL in krtt1c19e:lyn-tdtomato; krt4:lyn-egfp double transgenics. A–B’’’: Confocal images of the epidermis of krtt1c19e:lyn-tdtomato; krt4:lyn-egfp double transgenic larvae at 3 dpf (A–A’’’) and 7 dpf (B–B’’’) immunofluorescently stained for eGFP (green; A’, A’’’, B’, B’’’), tdTomato (red; A, A’’’, B, F’’’), ZO-1 (white; A’’–A’’’) and ΔNp63 (white; B’’, B’’’). The krtt1c19e promoter drives expression in the ΔNp63 positive basal layer. (TIF) [file pone.0084858.s004.tif]

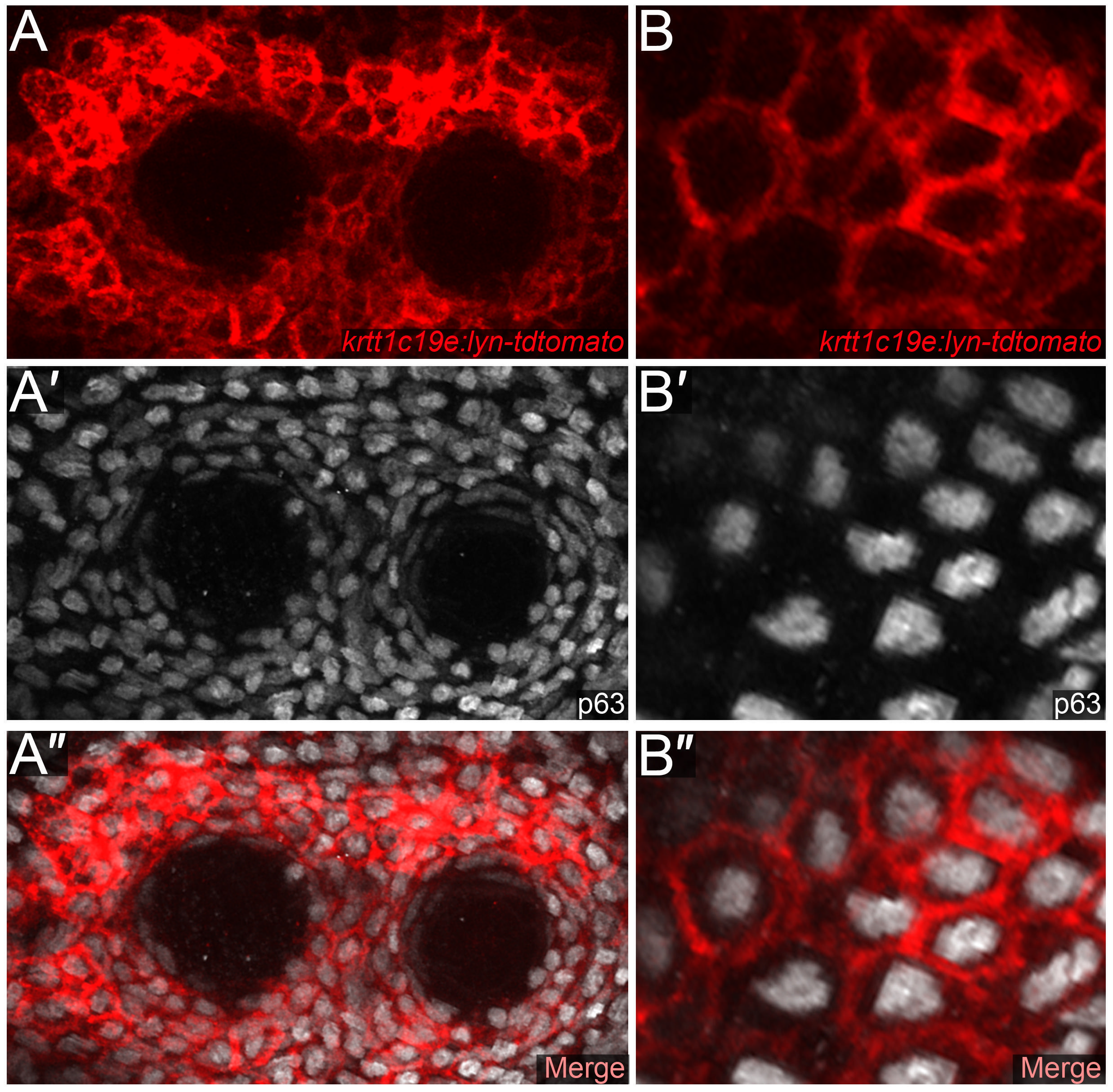

Supplement: Figure S5 — Strong expression of the krtt1c19e promoter in epidermal cells surrounding the adult neuromasts. Low (A–A”) and high (B–B”) magnification confocal images of cells surrounding trunk neuromasts of a krtt1c19e:lyn-tdtomato transgenic adult, immunofluorescently stained for tdTomato (red; A, B, A”, B”) and ΔNp63 (white; A’, B’, A”, B”). High level promoter activity is evident in p63 positive epidermal cells surrounding the neuromasts. (TIF) [file pone.0084858.s005.tif]
